# Nonalcoholic Fatty Liver Disease (NAFLD), But not Its Susceptibility Gene Variants, Influences the Decrease of Kidney Function in Overweight/Obese Children

**DOI:** 10.3390/ijms20184444

**Published:** 2019-09-09

**Authors:** Alessia Di Costanzo, Lucia Pacifico, Laura D’Erasmo, Luca Polito, Michele Di Martino, Francesco Massimo Perla, Ludovica Iezzi, Claudio Chiesa, Marcello Arca

**Affiliations:** 1 Department Internal Medicine and Medical Specialties, Sapienza University of Rome, 00161 Rome, Italy; derasmolaura@gmail.com (L.D.); luca.polito@uniroma1.it (L.P.); marcello.arca@uniroma1.it (M.A.); 2 Department of Pediatrics, Sapienza University of Rome, 00161 Rome, Italy; lucia.pacifico@uniroma1.it (L.P.); francescomassimo.perla@uniroma1.it (F.M.P.); ludovica_iezzi@yahoo.it (L.I.); 3 Department of Radiological Sciences, Sapienza University of Rome, 00161 Rome, Italy; micdimartino@hotmail.it; 4 Institute of Translational Pharmacology, National Research Council, 00133 Rome, Italy; claudio.chiesa@ift.cnr.it

**Keywords:** NAFLD, *PNPLA3* rs738409 gene polymorphism, renal function, overweight/obesity, children and adolescents

## Abstract

Nonalcoholic fatty liver disease (NAFLD) is associated with an increased risk of kidney disease in adults and children. However, it is uncertain whether this association is influenced by major NAFLD susceptibility genes. In a sample of 230 overweight/obese children, 105 with NAFLD (hepatic fat fraction ≥5% by magnetic resonance imaging) and 125 without NAFLD, rs738409 in *PNPLA3*, rs58542926 in *TM6SF2,* rs1260326 in *GCKR*, and rs641738 in *MBOAT7* were genotyped. Abnormal kidney function was defined as estimated glomerular filtration rate (eGFR) < 90 mL/min/1.73 m^2^ and/or the presence of microalbuminuria (24 h urinary albumin excretion between 30 and 300 mg). In comparison with children without NAFLD, those with NAFLD showed increased prevalence of reduced eGFR (13.3% vs. 1.6%; *p < 0.001*) and microalbuminuria (8.6% vs. 3.4%, *p = 0.025*). *TM6SF2, GCKR,* and *MBOAT7* risk alleles did not show any impact on kidney function, while the *PNPLA3* G allele was associated with lower eGFR, but only in children with NAFLD (*p* = 0.003). After adjustment for confounders, NAFLD (OR, 4.7; 95% CI, 1.5–14.8; *p*_adj_ = 0.007), but not the *PNPLA3* gene variant, emerged as the main independent predictor of renal dysfunction. Overall, our findings suggest that NAFLD remains the main determinant of decline in kidney function in overweight/obese children, while the *PNPLA3* rs738409 prosteatogenic variant has a small impact, if any.

## 1. Introduction

Nonalcoholic fatty liver disease (NAFLD) is the most common liver disease displaying an increasing prevalence in the general population mainly because of the concomitant rise in obesity and diabetes mellitus [1,2]. In addition to liver cirrhosis and hepatic carcinoma, NAFLD is also associated with a wide spectrum of extrahepatic complications including chronic kidney disease (CKD) [3]. Patients with NAFLD have a high prevalence of hypertension and diabetes mellitus, which are strongly associated with kidney function decline [4,5]. However, the possibility that the increase in hepatic fat content may, per se, play a role in favoring the extrahepatic consequences of NAFLD cannot be excluded [6,7]. To this regard, it remains to be determined whether genetic factors predisposing to NAFLD, with or without interacting with other risk factors, may play a role in these complications.

Indeed, several susceptibility gene variants predisposing to NAFLD have been consistently identified in different populations [8,9]. The most potent are the rs738409 in the *PNPLA3* and rs58542926 in the *TM6SF2* genes [9,10,11]. The rs1260326 in the *GCKR* and the rs641738 in the *MBOAT7* genes have also been reported to be associated with increased liver fat content, though less consistently [12,13]. However, their involvement in promoting deleterious effects on kidney function in NAFLD subjects has been less well established. Few reports have highlighted the potential influence of *PNPLA3* rs738409 and *TM6SF2* rs58542926 [14,15,16,17], but available data remain inconclusive. Yet, nothing is known about the effects of *GCKR* rs1260326 and *MBOAT7* rs641738 gene variants. Though we, and others, have reported that a genetic risk score (GRS) combining all these genetic variants may represent a strong predictor of the amount of liver fat content [18,19,20], its association with kidney function has not yet been tested. The demonstration of an independent role of the prosteatogenic genetic variants in favoring the deterioration of kidney function may not only highlight novel functions of these gene products, but it may also elucidate the damaging effects of an increased liver fat accumulation.

To this end, we studied a cohort of children at high risk of NAFLD, such as those presenting overweight/obesity, with the aims to enhance the potential effect of genetic variants and to determine their potential interaction with a common environmental condition associated with NAFLD. Furthermore, the determination of the role of prosteatogenic risk alleles in favoring the deterioration of kidney function in the early stages of life may be clinically relevant because of the alarming increasing prevalence of NAFLD in the pediatric population.

Therefore, in the present study we sought: 1) to determine whether Caucasian children and adolescents with overweight/obesity and NAFLD exhibit signs of renal functional alteration, as assessed by estimated glomerular filtration rate (eGFR) as well as by urinary albumin excretion, compared to those with overweight/obesity but without NAFLD; and 2) to assess whether *PNPLA3* rs738409, *TM6SF2* rs58542926, *GCKR* rs1260326, and *MBOAT7* rs641738 gene polymorphisms may influence renal function in overweight/obese youths with and without NAFLD.

## 2. Results

### 2.1. Characteristics of Study Population

Among study children, 105 (45.6%) met the criteria for NAFLD, while 125 (54.4%) were classified as non-NAFLD. A comparison of children with and without NAFLD is shown in Table 1. On average, NAFLD children were older; had higher waist circumference (WC), systolic BP, alanine aminotransferase (ALT), insulin, and homeostasis model assessment of insulin resistance (HOMA-IR); and lower concentrations of high-density lipoprotein cholesterol (HDL-C) (*p* < 0.05). The proportion of children with metabolic syndrome (MetS) was significantly higher in NAFLD than in the non-NAFLD group (26.7 % vs. 9.6%; *p* = 0.001).

In the entire cohort, 122 children (53.1%) were carriers of the *PNPLA3* G allele, 163 (70.9%) of the *MBOAT7* T allele, 183 (79.5%) of the *GCKR* T allele, and 32 (13.9%) of the *TM6SF2* T allele. Genotype distributions for any polymorphism followed a Hardy–Weinberg equilibrium (*p* > 0.05).

As expected, *PNPLA3* CG and GG genotypes were significantly more frequent in children with than in those without NAFLD (73% vs. 49%; *p* < 0.001) (Table 1). Similarly, the NAFLD group showed a higher percentage of children with *TM6SF2* CT + TT genotypes (*p* = 0.039). No differences were found in the prevalence of *GCKR* and *MBOAT7* variants according to the NAFLD status. In a multivariate model, the *PNPLA3* GG and *TM6SF2* CT + TT genotypes were, respectively, associated with a 14.9- and 3.1-fold increased risk of having NAFLD (data not shown). The GRS generated by using 3 risk alleles was highly predictive of NAFLD, even after adjustment for age, gender, body mass index (BMI), and HOMA-IR (OR, 7.7; 95% CI, 3.7–16.3; *p*_adj_ < 0.001) (data not shown).

### 2.2. Kidney Function According to Nonalcoholic Fatty Liver disease (NAFLD) Status

Compared to children without NAFLD, those with NAFLD showed significantly higher concentrations of 24 h urinary albumin excretion (*p* < 0.001), but they had similar mean values of eGFR (*p* = 0.27) (Table 1). However, a greater frequency of reduced eGFR (below 90 mL/min/1.73 m^2^) was observed in overweight/obese children with NAFLD compared to those without liver involvement (13.3 % vs. 1.6%; *p* < 0.001). The proportion of children with microalbuminuria was also higher in NAFLD compared to the non-NAFLD group (8.6 % vs. 3.4%; *p* = 0.025). None of the participants had eGFR <60 mL/min/1.73m^2^ or macroalbuminuria. As such, 21% of children with NAFLD had abnormal renal function as compared to 4% of children without NAFLD.

### 2.3. NAFLD Genetic Variants and Kidney Function

**Figure 1:**
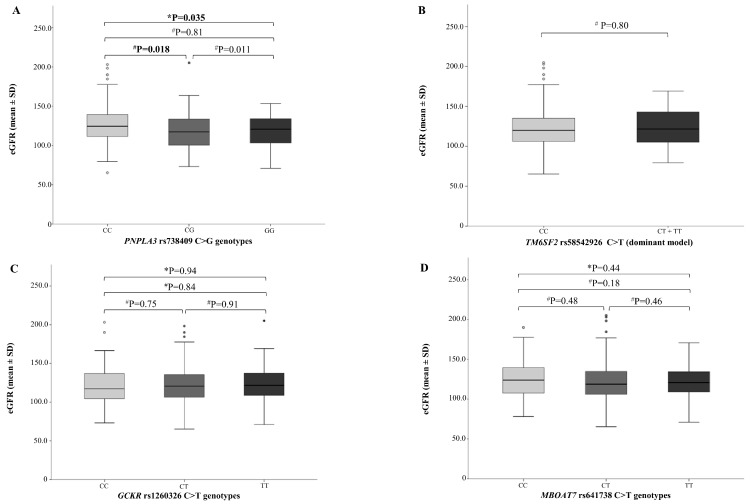
eGFR levels according to *PNPLA3*, *TM6SF2*, *GCKR*, and *MBOAT7* genotypes. eGFR is reported as mean ± SD, stratified by (**A**) *PNPLA3* rs738409 C>G genotype, (**B**) *TM6SF2* rs58542926 C>T genotype, (**C**) *GCKR* rs1260326 C>T genotype, and (**D**) *MBOAT7* rs641738 C>T genotype. A dominant model of inheritance was assumed for the *TM6SF2* rs58542926 polymorphism. * One-way ANOVA for comparisons between three groups; ^#^ Student’s *t*-test for comparisons between two groups.

After stratifying the entire cohort according to NAFLD susceptibility gene variants, mean values of eGFR significantly differed between *PNPLA3* rs738409 genotypes (Figure 1A; *p* = 0.035 for one-way ANOVA). Indeed, children carrying the *PNPLA3* G allele showed a small (6.3%) but significant decrease of eGFR as compared to wild-type carriers (126.7 ± 25.9 mL/min/1.73 m^2^ vs. 118.6 ± 22.5 mL/min/1.73 m^2^; *p* = 0.018). Conversely, no association between *TM6SF2, GCKR,* and *MBOAT7* gene variants and eGFR was detected.

**Figure 2:**
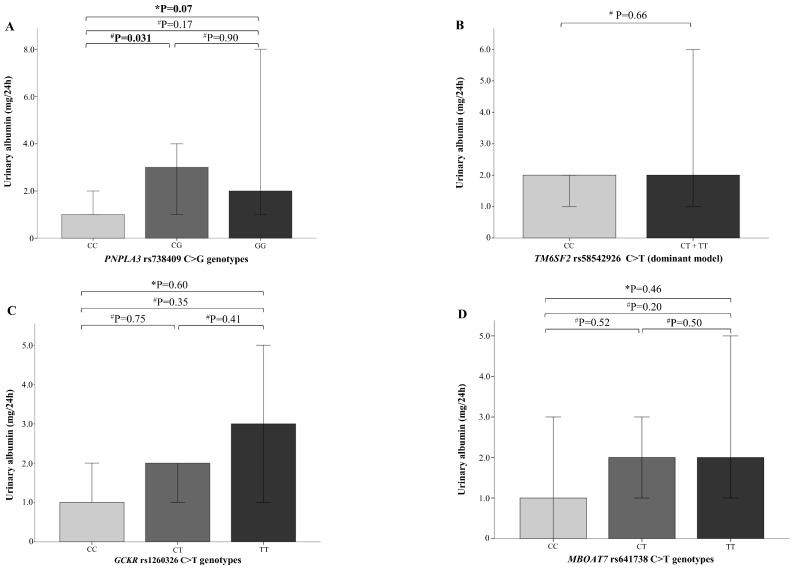
Levels of albuminuria (mg/24 h) according to *PNPLA3*, *TM6SF2*, *GCKR,* and *MBOAT7* genotypes. Urinary albumin is reported as median and interquartile ranges, stratified by (**A**) *PNPLA3* rs738409 C>G genotype, (**B**) *TM6SF2* rs58542926 C>T genotype, (**C**) *GCKR* rs1260326 C>T genotype, and (**D**) *MBOAT7* rs641738 C>T genotype. A dominant model of inheritance was assumed for the *TM6SF2* rs58542926 polymorphism. Kruskal–Wallis test for comparisons between three groups; ^#^ Mann–Whitney U test for comparisons between two groups.

Although heterozygous *PNPLA3* CG children showed increased median urinary albumin values (1 (0–3) mg/24 h vs. 3 (0–8.5) mg/24 h; *p* = 0.031), this effect was not consistent across *PNPLA3* genotypes (Figure 2A; *p* = 0.07). To test the potential combined effect of all variants on parameters of kidney function, we explored different options of weighted GRS. Linear regression analysis showed that the *PNPLA3* G allele was associated with the greatest variation of eGFR (R^2^ = 0.03; 95% CI, −14.5 to −2.02; β = −0.17; *p* = 0.01) and urinary albumin levels (R^2^ = 0.038; 95% CI, 0.008–0.394; β = 0.19; *p* = 0.041; data not shown). Based on these results, a dominant model of inheritance for estimating the effect of *PNPLA3* on kidney function was therefore considered.

Because in our cohort the rs738409 *PNPLA3* polymorphism was a determinant of NAFLD presence as well as reduced kidney function, we further explored a possible interaction between the *PNPLA3* G allele and the presence of NAFLD. To this aim, we compared the parameters of kidney function in the different genotypes according to the NAFLD status.

**Figure 3:**
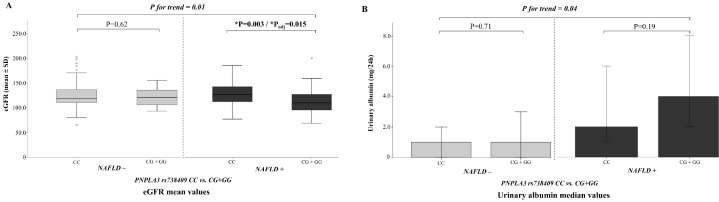
Association between *PNPLA3* genotypes and kidney function according to NAFLD status. (**A**) eGFR mean values and (**B**) urinary albumin median values, stratified by NAFLD status and *PNPLA3* CC vs. CG + GG genotypes. A dominant model of inheritance was assumed for the *PNPLA3* rs738409 polymorphism. NAFLD + and NAFLD - indicate children with and without NAFLD, respectively. (**A**) *p* for trend shows the linear association between eGFR mean values and *PNPLA3* CC vs. CG + GG genotypes independently of NAFLD status; * *p* =0.003 for comparisons between *PNPLA3* CC vs. CG + GG genotypes in NAFLD + children; * *p*_adj_ = 0.015 for comparisons between *PNPLA3* CC vs. CG + GG genotypes in NAFLD + children adjusted for BMI-SD score and hepatic fat fraction (HFF%). (**B**) *p* for trend shows the linear association between urinary albumin median values and *PNPLA3* CC vs. CG + GG genotypes independently of NAFLD status. Urinary albumin values have been log-transformed.

As shown in Figure 3A, children with NAFLD and the *PNPLA3* CG + GG genotype had 12% lower eGFR values than those with NAFLD and the *PNPLA3* CC genotype (115.6 ± 24.1 mL/min/1.73 m^2^ vs. 131.4 ± 25.3 mL/min/1.73 m^2^; *p* = 0.003). This difference persisted even after adjustments for BMI-SD score and hepatic fat fraction (HFF%) (*p*_adj_ = 0.015). No differences between *PNPLA3* genotypes were found when considering albuminuria in children with NAFLD (Figure 3B). Noteworthy, we found no significant effect of the *PNPLA3* G allele on eGFR values or albuminuria in children without NAFLD (Figure 3A,B).

Finally, we tested whether the *PNPLA3* G allele was an independent predictor of abnormal kidney function (e.g., eGFR < 90 mL/min/1.73 m^2^ and/or albuminuria ≥ 30 mg/24 h). As shown in Table S1, at univariate regression analysis, abnormal renal function was significantly associated with age, pubertal status, WC, diastolic BP, ALT levels, HFF%, and NAFLD presence in the entire cohort (all *p* < 0.05). In patients with NAFLD, abnormal renal function was significantly associated with age (*p* < 0.001) and pubertal status (*p* < 0.001), whereas in those without NAFLD it was associated with pubertal status (*p* < 0.05).

In a multivariate logistic regression model, after including age, pubertal status, gender, WC, and diastolic BP, only NAFLD, but not the *PNPLA3* G risk allele, emerged as a significant predictor of abnormal renal function in the whole cohort of children (OR, 4.7; 95% CI, 1.5–14.8; *p*_adj_ = 0.007) (Table 2). Notably, after further adjustments for the presence of MetS or HOMA-IR, results did not substantially change (data not shown).

## 3. Discussion

To our knowledge, this is the first report investigating the relationship between the four major NAFLD risk alleles in *PNPLA3*, *TM6SF2*, *GCKR,* and *MBOAT7* genes and kidney function in overweight/obese children with and without MRI-diagnosed NAFLD.

We demonstrated that: (a) obese children with NAFLD had a small but significant deterioration of kidney function when compared to those without NAFLD; (b) the *PNPLA3* rs738409 variant, but not those in *TM6SF2*, *GCKR*, and *MBOAT7* genes, was associated with mildly decreased eGFR levels and higher albuminuria; (c) the *PNPLA3* G allele had an impact on kidney function only in children with NAFLD; and (d) NAFLD, but not the *PNPLA3* G allele, emerged as the major independent predictor of abnormal renal function in obese children.

By considering a well-characterized population of 230 obese children and adolescents, we confirmed that NAFLD contributes to mild deterioration in kidney function. Indeed, 21% of NAFLD children showed abnormal renal function (of whom 13.3% had eGFR below 90 mL/min/1.73 m^2^, and 8.6% had microalbuminuria). These results are in line with other reports evaluating the prevalence of kidney dysfunction in children with NAFLD [16,17,21]. Though BMI-SD scores and duration of obesity have been reported to correlate with eGFR reduction in pediatric populations [22,23], in our study the effect of NAFLD on kidney function was found to be independent from BMI, as we enrolled only overweight/obese children. Several mechanisms have been proposed to account for the association between NAFLD and kidney dysfunction. They involve abnormal lipoprotein metabolism, altered intestinal barrier integrity and microbiome disturbance [24]. Moreover, recent human and experimental studies have also attributed obesity-related renal disease to ectopic lipid accumulation in the kidney (fatty kidney) [25,26]. Thus, as in our studyfat kidney content was not measured, future studies are warranted to clarify the issue.

We found that the prosteatogenic *PNPLA3* G allele showed a detrimental effect on eGFR levels, but this effect was very small and, more importantly, was detectable only in the NAFLD group. Similar investigations in the pediatric population are scanty and yield controversial results. Recently, in a cohort of 142 Caucasian children and adolescents with histologically confirmed NAFLD, Targher et al. [16] showed that homozygous M148M patients had lower eGFR values and higher 24 h proteinuria and stage 2 CKD (i.e., defined as eGFR value between 90 and 60 mL/min/1.73 m^2^) compared with other genotypes, even after adjustments for metabolic risk factors and severity of liver disease. However, 71% of them were carriers of the *PNPLA3* G allele, which modulates liver disease severity by favoring disease progression [27]. As such, 50% of these individuals carrying the 148M *PNPLA3* allele had definite non-alcoholic steatohepatitis (NASH). Thus, the high frequency of the *PNPLA3* rs738409 polymorphism in the cohort is likely to have increased the chances to find a positive association between the *PNPLA3* variant and unfavorable kidney function. Conversely, the frequency of *PNPLA3* genotypes in our cohort was well balanced and comparable to that observed in the general population; moreover, the inclusion of children with and without NAFLD provided an unbiased representation of the link between NAFLD, the *PNPLA3* variant, and kidney function.

An additional finding of the present study was the demonstration that prosteatogenic variants in *TM6SF2*, *GCKR*, and *MBOAT7* genes did not show any effect on kidney function. Also, by using alternative GRSs to measure the combined effect of NAFLD risk alleles, we excluded any involvement of *TM6SF2*, *GCKR*, and *MBOAT7* gene variants. This is the first study to test the involvement of *GCKR* and *MBOAT7* gene variants in the deterioration of kidney function in NAFLD subjects. The role of *TM6SF2* rs58542926 has been previously investigated only by Musso et al. [15] who reported a lower risk of renal damage in individuals carrying the 167K *TM6SF2* allele. However, since no renal function parameters were provided for the *TM6SF2* T carriers, it is unclear whether the reported association was restricted to NAFLD subjects or comprised the entire cohort.

We were able to evidence the greatest impact of fatty liver on the deterioration of kidney function. In fact, after adjustments for potential confounders, NAFLD, but not the *PNPLA3* G allele, emerged as being independently associated with eGFR < 90 mL/min/1.73 m^2^ and/or albuminuria > 30 mg/24 h, conferring a 4.5-fold risk of abnormal renal function.

Our findings are in line with the recent work by Marzuillo et al. [17] who studied a population of 591 obese children with and without NAFLD and with normal eGFR (> 90 mL/min/1.73 m^2^). The authors captured an effect of the *PNPLA3* I148M polymorphism on eGFR in NAFLD as well as in non-NAFLD groups, but the association was abolished in the latter group when potential confounders were taken into account.

This study presents some limitations. First, the cross-sectional design of the study precludes the establishment of a causal relationship between NAFLD and decline in kidney function. Second, the sample size of study children was relatively small. Third, we did not directly measure GFR (by plasma iohexol disappearance) to assess renal function. Nonetheless, we used the most widely accepted prediction formula for estimating GFR in the pediatric population (e.g., the bedside Schwartz formula).

In conclusion, we demonstrated that obese children and adolescents with MRI-diagnosed NAFLD are at risk for early renal dysfunction. No association was observed between the *TM6SF2*, *GCKR,* and *MBOAT7* genotypes and eGFR values as well as albuminuria. The small impact of the *PNPLA3* prosteatogenic variant on kidney function was restricted to children with NAFLD, but it disappeared when potential confounders were taken into account. It could be possible, therefore, that fatty liver and shared metabolic risk factors have a stronger effect on the decline of renal function than the *PNPLA3* G risk allele. Further studies are needed to better investigate the role of the I148M *PNPLA3* variant on the pathophysiological kidney activities.

## 4. Materials and Methods

### 4.1. Study Subjects

This cross-sectional study included 230 overweight (body mass index (BMI) > 85th and < 95th percentile for age and gender) or obese (BMI ≥ 95th percentile for age and gender) [19] Caucasian children and adolescents aged 6–16 years. They were consecutively recruited at the Hepatology Outpatient Clinic of the Department of Pediatrics, Sapienza University of Rome, Italy. Secondary causes of liver steatosis, including hepatic virus infections (hepatitis A–E and G, cytomegalovirus, and Epstein–Barr virus), autoimmune hepatitis, metabolic liver disease, α-1-antitrypsin deficiency, cystic fibrosis, Wilson’s disease, hemochromatosis, and celiac disease, were excluded using appropriate tests. Use of hepatotoxic drugs, as well as a history of type 1 or type 2 diabetes, smoking, and chronic alcohol intake were also exclusion criteria. None of the subjects had a history or known clinical, laboratory, and imaging signs of renal disease.

All study subjects received a complete physical examination including measurements of weight, standing height, BMI, waist circumference (WC), and systolic and diastolic blood pressure (BP), as reported in detail elsewhere [19]. The pubertal status was assessed according to the Tanner criteria. The degree of obesity was quantified using Cole’s least mean-square method, which normalizes the skewed distribution of BMI and expresses BMI as a standard deviation (SD) score [28]. The presence of metabolic syndrome (MetS) was determined as previously reported [19]. NAFLD was diagnosed by abdominal magnetic resonance imaging (MRI) (see below).

The study protocol was reviewed and approved by the Institutional Ethical Committee. Written, informed consent was obtained from the parents, caretakers, or guardians on behalf of the children enrolled in this study, in accordance with the principles of the Helsinki Declaration.

### 4.2. Laboratory Measurements

From all subjects, blood samples were taken in the morning after an overnight fast. All analyses were conducted by COBAS 6000 (Roche Diagnostics, Risch-Rotkreuz, Switzerland) [19,21]. Plasma insulin concentrations were measured on a COBAS e601 Module (Electrochemiluminescence Technology, Roche Diagnostics), and creatinine concentrations were measured by the kinetic colorimetric compensated Jaffé method using the Roche platform and the CREJ2–creatinine Jaffé Gen.2 assay (Roche Diagnostics, cod.0769282), which was standardized by isotope dilution mass spectrometry, traceable to National Institute of Standards and Technology creatinine standard reference material (SRM 914 and SRM 967) [29]. Urinary albumin was determined on 24 h urine collections by the turbidimetric immunoassay ALBT2 (Roche Diagnostics, cod.0767433). The remaining analytes were measured on a COBAS c 501 Clinical Chemistry Module (Photometric Technology, Roche Diagnostics) according to the manufacturer’s instructions.

Estimates of insulin resistance were calculated using the homeostasis model assessment of insulin resistance (HOMA-IR), defined by fasting insulin and fasting glucose [30].

### 4.3. Determination of Estimated Glomerular Filtration Rate (eGFR)

eGFR was estimated using the updated Schwartz formula (0.413 × height (cm)/serum creatinine (mg/dL)), which has been shown to be accurate for estimating GFR in the pediatric population [31]. A 24 h urinary protein excretion was also measured in 158 of the study subjects. In accordance with the Kidney/Dialysis Outcome Quality Initiative guidelines, eGFR categories were classified as follows: normal or high ≥90 mL/min/1.73 m^2^; mildly decreased, 60–89; mildly to moderately decreased, 45–59; moderately to severely decreased, 30–44; severely decreased, 15–29; and kidney failure <15 [32]. Microalbuminuria was diagnosed if the 24 h urinary albumin excretion rate was 30–299 mg, and macroalbuminuria if the 24 h albumin excretion rate was ≥300 mg [32].

### 4.4. Abdominal Magnetic Resonance Imaging (MRI).

Data were acquired on a 3.0 T MR scanner with a 50 mT/m maximum gradient length and 200 T/m/s maximum slew rate (Discovery MR 750; GE Medical Systems, Milwaukee, WI, USA) using an eight-element body torso-array coil system. Details of the scanning sequences have been reported elsewhere. The measurement of hepatic fat fraction (HFF%) have been done as previously described and validated [19,33,34]. The diagnosis of NAFLD was based on the detection of HFF ≥5% on MRI.

### 4.5. Genetic analysis.

Genomic DNA was extracted from whole blood according standard procedures. The rs641738 C>G (I148M) (*PNPLA3)*, rs58542926 C>T (E167K) (*TM6SF2),* rs1260326 C>T (L446P) (*GCKR),* and rs641738 C>T (G17E) (MBOAT7-TMC4) were considered for genotyping. Genotyping was performed in duplicate by TaqMan 5′-Nucleotidase assay [18,19,35,36]. The genetic risk score (GRS) was calculated based on the four selected SNPs, as previously described [18,19].

### 4.6. Statistical Analysis

Statistical analyses were performed by using the SPSS package (Version 22.0), SPSS Inc., Chicago, IL, USA. Data were reported as means and standard deviations for normally distributed variables or as median and interquartile range for non-normally distributed variables. Differences between the study groups were evaluated by *t*-tests or Mann–Whitney *U*-tests, ANOVA, or Kruskal–Wallis tests as appropriate. Proportions were compared by chi-squared and Fisher′s exact tests.

Genotype frequencies were assessed for Hardy–Weinberg equilibrium using the goodness-of-fit χ^2^ test. The four SNPs were tested using additive, dominant, and recessive genetic models. We also conducted a sensitivity analysis by calculating alternative GRS by excluding one genetic variant at a time to check for the robustness of associations between the four NAFLD-associated risk alleles and the decline of eGFR. The dominant model of inheritance for the *PNPLA3* gene variant was chosen as the best in our cohort to estimate associations with eGFR values, microalbuminuria, and the prevalence of abnormal renal function.

Association between the *PNPLA3* rs738409 variant, eGFR, and albuminuria (as a continuous measure) was tested by using an unadjusted linear regression model. Finally, multivariate logistic regression analysis was used to evaluate predictors of abnormal kidney function (i.e., defined as eGFR <90 mL/min/1.73 m^2^ and/or albuminuria ≥30 mg/24 h) in the entire cohort.

In this analysis, the presence of *PNPLA3* rs738409 CG + GG and CC genotypes were combined with covariates that emerged as potential confounding factors on the basis of their significance in univariate regression analyses or their biological plausibility. A *p* < 0.05 was considered statistically significant.

## Figures and Tables

**Table 1:** Comparison of overweight/obese children with and without nonalcoholic fatty liver disease (NAFLD).

	NAFLD (*n* = 105)	Without NAFLD (*n* = 125)	*p* Value
Age, years	11.0 ± 2.8	9.6 ± 2.7	<0.001
Male sex, %	64.8	50.4	0.028
BMI-SD score	2.0 ± 0.46	2.0 ± 0.49	0.86
Waist circumference, cm	90.6 ± 11.3	83.9 ± 10.9	<0.001
Systolic BP, mmHg	116 (110–120)	110 (105–118)	0.007
Diastolic BP, mmHg	66.5 (60.2–72.5)	65.0 (60–70)	0.05
Total cholesterol, mg/dL	156.7 ± 32.7	163.6 ± 31.05	0.10
HDL-C, mg/dL	47.9 ± 11.2	52.1 ± 13.5	0.013
Triglycerides, mg/dL	77 (55–119)	70 (50–96)	0.10
AST, U/L	24 (20–30)	23 (20–26)	0.10
ALT, U/L	27 (18–36)	17 (15–22)	<0.001
Glucose, mg/dL	83 (78.1–86.6)	83 (77.0–86.0)	0.95
Insulin, µU/mL	14.05 (9.7–20.7)	10.6 (7.5–14.4)	<0.001
HOMA-IR	2.7 (1.9–4.3)	2.1 (1.5–2.9)	0.002
HbA1c, %	5.1 (4.8–5.3)	5.2 (4.8–5.3)	0.69
Hepatic fat fraction, %	10.0 (7.0–19.5)	1.0 (0–2.0)	<0.001
MetS, %	26.7	9.6	0.001
eGFR, mL/min/1.73 m^2^	120.4 ± 25.4	123.9 ± 23.2	0.27
Urinary albumin, mg/24 h *	3 (1–11)	1 (0–3)	<0.001
eGFR < 90 mL/min/1.73 m^2^, *n* (%)	14 (13.3)	2 (1.6)	<0.001
Microalbuminuria, *n* (%) *	9 (8.6)	3 (3.4)	0.025
eGFR < 90 mL/min/1.73 m^2^ and/or microalbuminuria, *n* (%)	22 (21)	5 (4.0)	<0.001
*PNPLA3* rs738409			<0.001
C/C	32 (30.5)	76 (60.8)	
C/G	55 (52.4)	45 (36.0)	
G/G	18 (17.1)	4 (3.2)	
*TM6SF2* rs58542926			0.039
*C/C*	84 (80.0)	114 (91.2)	
*C/T*	20 (19.0)	11 (8.8)	
*T/T*	1 (1.0)	-	
*GCKR* rs1260326			0.28
*C/C*	19 (18.1)	28 (22.4)	
*C/T*	53 (50.5)	69 (55.2)	
*T/T*	33 (31.4)	28 (22.4)	
*MBOAT7* rs641738			0.12
*C/C*	36 (34.3)	31 (24.8)	
*C/T*	40 (38.1)	64 (51.2)	
*T/T*	29 (27.6)	30 (24.0)	

Results are expressed as *n* (%), mean ± SD, or median (25th–75th interquartile range). * Of the 158 patients assessed for 24 h urinary albumin, there were 70 patients with NAFLD and 88 without NAFLD. BMI-SD score, Body mass index-standard deviation score; HDL-C, high-density lipoprotein cholesterol; AST, aspartate aminotransferase; ALT, alanine aminotransferase; HOMA-IR, homeostasis model assessment of insulin resistance; MetS, metabolic syndrome; eGFR, estimated glomerular filtration rate.

**Table 2:** Multivariate analysis of variables associated with eGFR < 90 mL/min/1.73 m^2^ and/or albuminuria in the entire study population.

	Odds Ratio (95% CI)	Adjusted *p* Value
Age, years	1.1 (0.8–1.5)	0.65
Male gender	1.2 (0.4–3.6)	0.70
Pubertal status	1.6 (0.9–3.0)	0.09
Waist Circumference	1.0 (0.9–1.05)	0.92
Diastolic BP	1.0 (0.9–1.08)	0.74
NAFLD	4.7 (1.5–14.8)	0.007
*PNPLA3* (dominant model)	0.9 (0.4–3.2)	0.85

Included in the model were age, gender, pubertal status, and variables significantly associated with abnormal renal function such as waist circumference (WC), diastolic BP, NAFLD, and *PNPLA3* (dominant model).

## Supplementary Materials

Supplementary materials can be found at https://www.mdpi.com/1422-0067/20/18/4444/s1.
