# Supplementary material for: Nonalcoholic Fatty Liver Disease (NAFLD), But not Its Susceptibility Gene Variants, Influences the Decrease of Kidney Function in Overweight/Obese Children
Source: Int J Mol Sci. 2019 Sep 9;20(18):4444. doi: 10.3390/ijms20184444 (PMC6769859; doi:10.3390/ijms20184444)
Supplement: Supplementary file 1 [file ijms-20-04444-s001.pdf]

Table S1. Univariate regression analysis of variables* associated with eGFR<90 mL/min/1.73 m^2 and/or microalbuminuria in the entire cohort, and according to NAFLD status.

**Entire cohort**	**Odds ratio (95% CI)**	**P value**
Age, years	1.42 (1.22-1.66)	<0.001
Pubertal status, %	2.12 (1.57-2.88)	<0.001
Waist Circumference, cm	1.05 (1.02-1.09)	0.002
Diastolic blood pressure, mmHg	1.05 (1.00-1.11)	0.03
ALT, U/L	1.01 (1.00-1.02)	0.018
Hepatic fat fraction, %	1.04 (1.01-1.07)	0.007
NAFLD	6.36 (2.32-17.5)	<0.001
PNPLA3 rs738409 (dominant model)	2.30 (0.96-5.51)	0.06
**Children with NAFLD**	**Odds ratio (95% CI)**	**P value**
Age, years	1.40 (1.16-1.68)	<0.001
Pubertal status, %	1.94 (1.37-2.47)	<0.001
**Children without NAFLD**	**Odds ratio (95% CI)**	**P value**
Pubertal status, %	2.13 (1.08-4.17)	0.028

* Only significant associations were reported.
